# Impact of seminal trace element and glutathione levels on semen quality of Tunisian infertile men

**DOI:** 10.1186/1471-2490-12-6

**Published:** 2012-03-19

**Authors:** Fatma Atig, Monia Raffa, Ben-Ali Habib, Abdelhamid Kerkeni, Ali Saad, Mounir Ajina

**Affiliations:** 1 Unit of Reproductive Medicine, University Farhat Hached Hospital, 4000 Soussa, Tunisia; 2 Research Laboratory of "Trace elements, free radicals and antioxidants", Biophysical Department, Faculty of Medicine, University of Monastir, 5000 Monastir, Tunisia; 3 Department of Cytogenetic and Reproductive Reproduction, Farhat Hached, University Teaching Hospital, 4000 Sousse, Tunisia

**Keywords:** Antioxidants, Idiopathic oligoasthenoteratozoospermia, Male infertility, Oxidative stress, Reactive oxygen species, Spermatozoa, Seminal plasma

## Abstract

**Background:**

Growing evidence indicates that oxidative stress can be a primary cause of male infertility. Non-enzymatic antioxidants play an important protective role against oxidative damages and lipid peroxidation. Human seminal plasma is a natural reservoir of antioxidants. The aim of this study was to determine glutathione (GSH) concentrations, trace element levels (zinc and selenium) and the lipid peroxidation end product, malondialdehyde (MDA), in the seminal plasma of men with different fertility potentials.

**Methods:**

Semen samples from 60 fertile men (normozoospermics) and 190 infertile patients (74 asthenozoospermics, 56 oligozoospermics, and 60 teratozoospermics) were analyzed for physical and biochemical parameters. Zinc (Zn) and selenium (Se) levels were estimated by atomic absorption spectrophotometry. Total GSH (GSHt), oxidized GSH (GSSG), reduced GSH (GSHr) and MDA concentrations were measured spectrophotometrically.

**Results:**

Zn and Se concentrations in seminal plasma of normozoospermics were more elevated than the three abnormal groups. Nevertheless, only the Zn showed significant differences. On the other hand, Zn showed positive and significant correlations with sperm motility (P = 0.03, r = 0.29) and count (P < 0.01, r = 0.49); however Se was significantly correlated only with sperm motility (P < 0.01, r = 0.36). GSHt, GSSG and GSHr were significantly higher in normozoospermics than in abnormal groups. We noted a significant association between seminal GSHt and sperm motility (P = 0.03). GSSG was highly correlated to sperm motility (P < 0.001) and negatively associated to abnormal morphology (P < 0.001). GSHr was significantly associated to total sperm motility (P < 0.001) and sperm count (P = 0.01). MDA levels were significantly higher in the three abnormal groups than in normozoospermics. Rates of seminal MDA were negatively associated to sperm motility (P < 0.01; r = -0.24) and sperm concentration (P = 0.003; r = -0.35) Meanwhile, there is a positive correlation between seminal lipid peroxidation and the percentage of abnormal morphology (P = 0.008).

**Conclusions:**

This report revealed that decreased seminal GSH and trace element deficiencies are implicated in low sperm quality and may be an important indirect biomarker of idiopathic male infertility. Our results sustain that the evaluation of seminal antioxidant status in infertile men is necessary and can be helpful in fertility assessment from early stages.

## Background

It is increasingly recognized that reactive oxygen species (ROS) are of significant pathophysiological importance in the etiology of male infertility [1]. ROS are highly reactive oxidizing agents belonging to the class of free radicals containing one or more unpaired electrons which are continuously being generated through metabolic and pathophysiologic processes [2]. It has been postulated that oxidants interfere with normal sperm function via membrane lipid peroxidation and fragmentation of nucleic acids, which result in sperm dysfunction [3]. Due to their abundance of membrane polyunsaturated fatty acids (PUFAs) and their capacity to generate ROS, human spermatozoa are highly susceptible to oxidative stress [3]. The more important marker of lipid peroxidation is MDA. This by-product has been used in biochemical assays to monitor the degree of peroxidative damage sustained by spermatozoa. The results of such assay exhibit an excellent correlation with the degree to which sperm function is impaired [4]. High levels of seminal MDA represent increased lipid peroxidation rates, which diminishes fertility [3,4].

Hence, human spermatozoa are known to possess all of the major antioxidant defensive systems' including catalase, superoxide dismutase (SOD), glutathione peroxidase (GPX) and glutathione reductase (GRD) [5], their effectiveness is impaired by their limited concentrations and distribution. Interestingly, the seminal plasma is well equipped with an array of antioxidant defence mechanisms to protect the spermatozoa against oxidants. Antioxidants that are present in the seminal plasma compensate for the deficiency in cytoplasmic enzymes in the spermatozoa [4]. The activities of seminal antioxidant enzymes like SOD and GPX, has been measured in several studies [5]. Additionally, low molecular weight scavengers from seminal plasma appeared to be more important than enzymes, for example, trace elements and GSH [1,5].

Trace elements in human semen comprising Zn and Se have been shown to be essential for testicular development and spermatogenesis [6-9]. Zn is one of the primary factors responsible for deoxyribonucleic acid (DNA) transcription and protein synthesis which are major parts of sperm development [8]. Its concentrations are very high in the male genital organs, particularly in the prostate gland [7]. Zn can oppose the oxidation by binding sulphydryl groups in proteins and by occupying binding sites for copper in lipids and DNA [8]. It can also be a cofactor of Copper/Zn-SOD [6]. Recent studies hypothesized that insufficient intake of Zn impairs antioxidant defences. This may be an important risk factor in oxidant release and makes the spermatozoa more susceptible to lipid peroxidation [6].

Se, in particular, is an essential element for normal testicular development, spermatogenesis, and spermatozoa motility and function [9]. Se may protect against oxidative DNA damage in human sperm cells. However, the exact mechanism by which Se eliminates oxidative stress to improve male fertility and semen quality in humans is still controversial. The role of Se could be mediated via selenoenzymes, such as GPX [10]. The best-characterized spermatozoal effects of Se deficiency are: important loss of motility, breakage at the midpiece level [9,10] and increased incidence of sperm-shape abnormalities, mostly of the sperm head [10].

One more important antioxidant in humans is GSH [3]. It serves as a cofactor for GPX and reacts directly with ROS by its free sulphydryl groups. When present in extracellular space, GSH is able to react directly with cytotoxic aldehydes produced during lipid peroxidation and thus protects the sperm plasma membrane [11]. GSHt is an imperative antioxidant via its ability to cycle between GSHr and GSSG. GSHt scavenges excess ROS and oxidized to GSSG, which is subsequently converted back to GSHr via GRD. The fertility promoting role of GSH has been established through different studies where GSH has been found to be reduced in oligozoospermics and azoospermics compared to normozoospermics [12].

Keeping in view the main protection provided by seminal plasma antioxidants against oxidative damages, the purpose of our present study was to (1) evaluate the non-enzymatic antioxidant profiles of idiopathic infertile men and to (2) assess their impact on semen parameters. In order to fulfil this goal, we measured the concentrations of Zn, Se, GSH and MDA in the seminal plasma of 250 infertile men. To our knowledge, this report which investigated the repercussions of human seminal antioxidants on sperm quality constitutes the first one conducted in Tunisia.

## Methods

### Study design and subject selection

A prospective controlled study was employed, involving 250 men (22-50 years) consulting for infertility evaluation in our laboratory of Cytogenetic and Reproductive Biology, Farhat Hached University Hospital, Sousse (Tunisia). Inclusion and exclusion criteria for selection of idiopathic infertile males were as follows:

#### Inclusion criteria

Males of couples living together with regular unprotected coitus for a reasonable period of time but not less than 1 year without conception.

#### Exclusion criteria

A detailed medical history was performed for all studied cases. Subjects currently on any medication or antioxidant supplementation were not included. In addition, subjects with testicular varicocele, genital infection, leukocytospermia, chronic illness and serious systemic diseases, smokers and alcoholic men were excluded from the study because of their well-known high seminal ROS levels and decreased antioxidant activity.

#### Study consent

A written consent of each subject was taken after explaining the aims and objectives of the study and its benefits on individual and society. Also, the study was approved by the Local Ethic Committee of the Farhat Hached University Hospital, Sousse (Tunisia).

### Semen analysis

Semen samples were collected by masturbation into sterile cups following 3 days of sexual abstinence. After 30 minutes of liquefaction, standard semen parameters (Volume, motility, concentration and morphology) were immediately evaluated according to the World Health Organization guidelines (1999) [13]. Sperm motility was classified into four categories: rapid progressive motile (Type a), slow progressive motile (Type b), non-progressive motile and immotile spermatozoa, and was assayed at exactly 0.5 and 2 hours after liquefaction.

Total progressive motility was defined as the combination of type a rapid motility and type b slow progressive (At least 30% of sperm should have normal motility (categories a + b)). Sperm concentration was determined with an improved Neubauer Hemacytometer^® ^counting chamber and normal values of concentration were " > 20 × 106 sperm/ml". Morphology was measured by recording the percentage of abnormal forms in the sample based on the classification of "David" [14] and at least 30% of sperm should have normal morphology.

After semen analysis, selected subjects were divided into four categories (Details of groups and semen characteristics were described in Tables 1 and 2):

**Table 1:** General characteristics of the studied population

parameters	Characteristics	Number(n)	Percentage (%)
Age (Years)	≤ 35	105	42
	> 35	145	58

Duration of infertility	≤ 10	183	72.2
	> 10	67	28.8

Type of infertility	Primary	191	76.4
	Secondary	59	32.6

Area	Rural	94	37.6
	Urban	156	62.4

Diet	Vegetarian	138	55.2
	Mixed	112	54.8

Sperm criteria	Fertile	60	24
	Asthenozoospermics	74	29.6
	Oligozoospermics	56	22.4
	Teratozoospermics	60	24

Duration of sexual abstinence (Days)	3	250	100

**Table 2:** Descriptive statistics and comparison of conventional semen parameters fertile and the three infertile groups

Parameters	Fertile (n = 60)	Astheno (n = 74)	Oligo (n = 56)	Terato (n = 60)	P-Value		
					
					Fertile Vs Astheno	Fertile Vs Oligo	Fertile Vs Terato
**Age (years)**	33.43 ± 4.40	34.95 ± 4.20	40.27 ± 4.50	39.00 ± 5.01	NS	NS	NS
**Mean ± SD Min-Max**	22.00-41.00	29.00-42.00	30.00-50.00	28.00-50.00			

**Volume (ml)**	3.89 ± 1.48	3.34 ± 1.67	4.21 ± 2.44	3.65 ± 1.73	NS	NS	NS
**Mean ± SD Min-Max**	2.00-5.56	2.00-7.58	2.00-8.23	2.00-6.34			

**Sperm motility (%)**	48.72 ± 14.22	18.40 ± 9.62	45.72 ± 13.42	44.32 ± 12.45	**P < 0.001**	NS	NS
**Mean ± SD Min-Max**	43.00-100	1.00-30.00	40.00-99.00	40.00-97.00			

**Sperm Count (x10^6^/ml)**	75.86 ± 23.83	46.21 ± 24	9.56 ± 6.51	54.00 ± 26.23	**P < 0.001**	**P < 0.001**	**0.001**
**Mean ± SD Min-Max**	30.00-100	20.00-100	1.00-15.00	26.00-100			

**Abnormal morphology (%)**	60.90 ± 13.28	60.42 ± 11.85	65.24 ± 16.36	81.79 ± 11.49	NS	NS	**P < 0.001**
**Mean ± SD Min-Max**	39.00-65.00	41.00-72.00	41.00-69.00	77.00-98.00			

Note: Astheno = Asthenozoospermics, Oligo = Oligozoospermics, Terato = Teratozoospermics, Max = maximum, Min = minimum, ml = milliliter, SD = Standard Deviation. Data are expressed as means ± SD. NS = Not significant. P ≤ 0.05 = significant.

a. **Normozoospermics-Normo [60 cases]: **Subjects of this group represent fertile men (controls). They are characterized by normal values for sperm motility (> 30%), sperm concentration (> 20 × 106 sperm/ml) and normal sperm morphology (> 30%).

b. **Asthenozoospermics-Astheno [74 cases]: **This study group consists of isolated asthenozoospermic men whose infertility is determined mainly by less than normal levels for sperm motility (< 30%), with normal values of morphologically normal sperm (< 30%) and sperm concentration (> 20 × 106 sperm/ml).

c. **Oligozoospermics-Oligo [56 cases]: **Infertility of this group is defined mainly by an isolated oligozoospermia (< 20 × 106 sperm/ml), with normal levels for sperm motility (> 30%) and morphologically normal sperm (> 30%)

d. **Teratozoospermics-Terato [60 cases]: **This category of patients consists of isolated teratozoospermic men whose infertility is determined essentially by less than normal levels for morphologically normal sperm (< 30%), with normal values of sperm concentration (> 20 × 106 sperm/ml) and motility (> 30%).

After semen analysis, seminal plasma was separated from spermatozoa by centrifugation at 3500 rpm for 15 minutes and stored at -80°C until antioxidant analysis.

### Chemicals

All reagents and chemicals were of analytical grade or higher purity and obtained from standard commercial suppliers. Ultra-pure water was received from water purification Milli-Q system (Millipore Corporation, USA).

### Biochemical Procedures

a. **Analytical methods for determination of Zn and Se levels**

For all the experiment, flame atomic absorption spectrophotometry (AAS) was adopted for Zn determination and furnace AAS for Se measurement. The measures were implemented using a Zeenit 700-Analytik-Jena, Germany, equipped with deuterium and Zeeman background correction, respectively, as recommended by the manufacturer. Detection limits for these microelements were as follows: Zn (0.47 μg/l) and Se (0.78 μg/l).

b. **Determination of GSHt and GSSG contents**

GSHt and GSSG levels were measured spectrophotometrically in deproteinized seminal plasma samples, by the method of Akerboom & Sies, (1981) [15], using 5,5'-dithiobis (2-nitrobenzoic acid). Absorbance values were compared with standard curves from known amounts of GSH standards.

c. **Assessment of oxidative stress by measurement of Lipid Peroxidation**

Lipid peroxidation was measured by determining the MDA production, using the Thiobarbituric Acid (TBA) method (Yagi, (1976)) [16]. After liquefaction, semen samples were centrifuged at 3500 rpm for 15 min to get seminal plasma. Then, 0.1 ml of seminal plasma was added to 0.75 ml of TBA reagent (0.8 g of 2-TBA dissolved in 80 ml of distilled water with 0.5 ml of NaOH. Perchloric acid (7%) was added to this mixture in order to adjust the pH = 7.4). This mixture was heated for 1 hour at 95°C in a warm water bath. After cooling, the tube was centrifuged for 10 min at 4000 rpm and the supernatant's absorbance was read on a spectrophotometer at 535 nm.

### Statistical analysis

The Windows computing program Statistical Package for the Social Sciences "SPSS 11.0" (SPSS, Chicago, IL, USA) was used for analyzing the data. All data were expressed as mean ± standard deviation (S.D.). The differences that existed between the evaluated study groups were assessed by performing an analysis of variance (One-way ANOVA) and the post hoc test (Tukey test) to conduct pair-wise comparisons. The differences were considered to be significant at values of P ≤ 0.05. Finally, Pearson's correlation was performed to examine the correlation between trace elements, MDA and semen quality. All hypothesis testing were two-sided with a probability value of 0.05 deemed as significant (P < 0.05 = significant*; P < 0.001 = highly significant**).

## Results

As shown in Table 2 the mean of age of the fertile group (33.4 ± 4.4 years) was decreased when compared to asthenozoospermics (34.9 ± 4.2 years), oligozoospermics (40.2 ± 4.5 years) and teratozoospermics (39 ± 5 years), but the difference was not significant. There was no significant correlation between patient's age and seminal antioxidant status. The mean values of the examined sperm parameters in controls and infertile groups are shown in Table 2. Sperm count, motility and the percentage of normal morphology were significantly higher in normozoospermics than the three abnormal groups.

### Trace element and GSH levels

In this study and as shown in Table 3, concentrations of seminal trace elements "Zn and Se" were more elevated in the control group than the three abnormal groups. However, only the Zn showed significantly differences after comparison between controls and asthenozoospermics (P = 0.02), oligozoospermics (P = 0.008) and teratozoospermics (P = 0.04). Moreover, a significant increase of mean GSHt were found in normozoospermics (57.00 ± 19.54 μmol/l) compared to asthenozoospermics (38.41 ± 23.92 μmol/l P < 0.001) and teratozoospermics (45.77 ± 17.62 μmol/l P = 0.002). Also, a highly significant decline in GSSG concentration was detected in the seminal plasma of teratozoospermics compared to the fertile group (P < 0.001) (Table 3). GSHr showed an increased concentration in normozoospermic group compared to Asthenozoospermics (P < 0.001); however, there was no significant difference observed when compared with oligozoospermics and teratozoospermics.

**Table 3:** Seminal antioxidant status and MDA concentrations of the different studied groups

Parameters	Fertile (n = 60)	Astheno (n = 74)	Oligo (n = 56)	Terato (60)		P-Value	
				
					Fertile Vs. Astheno	Fertile Vs. Oligo	Fertile VS. Terato
**Zinc (mg/l)**	144 ± 42.13	122 ± 34.69	120.51 ± 25.33	126 ± 24.82	**0.02**	**0.008**	**0.04**
**Mean ± SD Min-Max**	49.00-239	54.00-190	46.52-94.55	52-200			

**Selenium (μg/l)**	64 ± 20.64	56.00 ± 22.81	55.00 ± 21.44	57.00 ± 24.31	NS	NS	NS
**Mean Min-Max**	44.00-84.00	30.00-72.00	41.00-69.00	36.00-76.00			

**GSHt (μmol/l)**	57.00 ± 19.54	38.41 ± 23.92	52.86 ± 7.08	45.77 ± 17.62	**< 0.001**	NS	**0.002**
**Mean ± SD Min-Max**	25.63-99.55	7.18-96.84	43.88-60.86	14.22-68.45			

**GSSG (μmol/l)**	22.81 ± 13.38	21.89 ± 12.67	24.84 ± 11.75	9.74 ± 5.82	NS	NS	**< 0.001**
**Mean ± SD Min-Max**	0.00-55.19	2.63-79.07	8.82-43.86	2.27-35.53			

**GSHr**	34.27 ± 14.91	16.63 ± 12.83	28.00 ± 14.22	36.00 ± 16.71	**< 0.001**	**NS**	NS
**Mean ± SD Min-Max**	19.00-55.00	13.00-31.00	13.85-43.00	19.00-42.00			

**MDA (μmol/l)**	2.30 ± 0.94	3.52 ± 1.93	3.21 ± 1.37	3.64 ± 1.73	**0.002**	**0.004**	**< 0.001**
**Mean ± SD Min-Max**	1.37-2.33	2.21-4.83	2.28-4.29	2.66-4.68			

Note: Astheno = Asthenozoospermics, Oligo = Oligozoospermics, Terato = Teratozoospermics, GSHt = Total glutathione, GSSG = Oxidized glutathione, GSHr = reduced glutathione, MDA = Malondialdehyde acid, Max = maximum, Min = minimum, ml = millilitre, SD = Standard Deviation, NS = Not significant. Data are expressed as means ± SD. P ≤ 0.05 = significant.

### Seminal MDA content

Lipid peroxidation as expressed by MDA level in the seminal plasma was found to be significantly higher in the three abnormal groups than in fertile group. Mean MDA levels in abnormal groups were found to be 3.52 ± 1.93 μmol/l in the asthenozoospermic group,

3.21 ± 1.37 μmol/l in the oligozoospermic group and 3.64 ± 1.73 μmol/l in the teratozoospermic group (Table 3). In effect, rates of seminal MDA were negatively associated to sperm motility (P < 0.01; r = -0.24) and sperm concentration (P = 0.003; r = -0.35) Meanwhile, there was a positive correlation between seminal lipid peroxidation and the percentage of abnormal morphology (P = 0.008; r = 0.19) (Table 4).

**Table 4:** The value of Pearson's correlation coefficients calculated between the antioxidant parameters and sperm criteria

Elements	Motility	Sperm count	Atypical forms
Zinc	0.29*	0.49**	NS

Selenium	0.36**	NS	NS

GSHt	0.13*	NS	NS

GSSG	0.22**	NS	-0.39*

GSHr	0.42**	0.11*	**NS**

MDA	-0.24*	-0.35*	0.19*

Note: GSHt = Total glutathione, GSSG = Oxidized glutathione, MDA = Malondialdehyde acid, NS = Not significant. * Significant correlation P < 0.05, ** Correlation strongly significant P ≤ 0.001.

### Correlations between seminal antioxidant status and sperm parameters

Significant positive correlations were detected between seminal Zn concentrations and sperm motility (P = 0.03, r = 0.29) and sperm concentration (P < 0.01, r = 0.49). Seminal Se showed a highly significant relationship only with sperm motility (P < 0.01, r = 0.36) (Table 4).

As shown in Table 4 we noted a significant association between seminal GSHt and sperm motility (P = 0.03, r = 0.13). However, seminal GSSG was highly correlated to sperm motility (P < 0.001, r = 0.22) and negatively associated to the percentage of sperm atypical forms (P < 0.001, r = -0.39) Seminal GSHr was significantly associated to sperm motility (P < 0.001, r = 0.42) and sperm concentration (P = 0.01, r = 0.11).

## Discussion

The key results in this study were: ***(1) ***An impaired antioxidant status was observed in seminal plasma of infertile men. ***(2) ***The fertile group showed a significant decrease in seminal lipid peroxidation levels compared to the three abnormal groups. ***(3) ***Positive correlations exist between enhanced seminal antioxidant status and good semen quality. Total sperm motility was significantly correlated to the seminal trace elements and different forms of GSH. The sperm concentration was positively associated to the seminal Zn and seminal GSHr amounts. However, the percentage of atypical sperm forms showed a significantly negative relationship to seminal GSSG. ***(4) ***Inversely to the non-enzymatic antioxidants, seminal MDA levels were negatively correlated to the sperm parameters. These findings indicated that some idiopathic infertile men may be poorly equipped to deal with oxidative stress due to the impaired seminal antioxidant defences. As a consequence, we suggest such a possibility and call for more systematic research on the role of oxidative stress in idiopathic male infertility.

The detailed biochemical mechanisms underlying the physiopathology of male infertility are not clearly understood. There has been evidence supporting the notion that major changes in seminal antioxidants could be related to abnormal spermatozoa function and fertilization capacity [9]. Hence, seminal plasma is considered to be the central source of antioxidants that protect sperm cells against oxidative damages. The antioxidant system eliminates ROS to maintain a reduced environment in cells through enzymatic and non-enzymatic approaches. The most studied antioxidants are the SOD, GPX and catalase enzymes. Information about seminal non-enzymatic antioxidants and male fertility potential is quite inadequate till date. Moreover, it is important to underline the contradictions and the controversial outcomes found in the literature. In fact, these differences can be due to several variables among which are inclusion and exclusion criteria for patient selection, analytical methodologies, sperm anomalies (in our study, each patient group has a single sperm anomaly; whether asthenozoospermia, or oligozoospermia or teratozoospermia), lifestyle or dietary pattern and the patient's origin.

In our study, we tried to explore the concentrations of Zn, Se and GSH in the seminal plasma of our patient groups and controls in order to estimate their contribution to maintain a good quality of sperm.

The mean seminal Zn concentrations in control and abnormal groups were between 120 and 144 mg/l. These values were comparable with a few similar studies reported by Lewis Jones et al. (118 mg/l) [17] and Kruse et al. (123 mg/l) [18]. However, the reported Zn levels in other studies were considerably higher than our findings; Omu et al. (171 mg/l) [19] and Chia et al. (183 mg/l) [7]. These differences may be due to the slightly different techniques used in the measurement of Zn concentrations and the difference in cigarette-smoking habits. In comparison with the control group, our infertile patients showed decreased seminal Zn concentrations; however these differences were not significant. Inflammatory conditions, prostatitis, accumulation of toxic heavy metals and frequent ejaculation can negatively influence the secretory function of the prostate and lead to a drop in seminal Zn content [7,20]. Nevertheless, these factors were excluded from our investigation and reduced Zn amounts of infertile patients can be explained by other mechanisms. Increased sperm ROS in infertile men explain the decrease of seminal Zn concentrations, arising the harmful effects of ROS to sperm cells which are associated with abnormal sperm parameters [8]. Powell [21] supports this hypothesis, noting that reduction in Zn concentration can lead to an increase in oxidation of membrane lipids, DNA and proteins; which can provoke the loss of membrane integrity. In our study, the negative and not significant correlation between Zn and MDA confirmed that reduction in seminal Zn levels is associated to the decline of its antioxidant capacity and the increase of lipid peroxidation and low sperm quality.

Additionally, seminal Zn concentrations demonstrated significant differences between fertile and infertile men, which corroborated with the findings of Colagar et al. [6]. Furthermore, like Zaho et al. [22] we observed positive relationships between good sperm production, motility and increased seminal Zn content. With these findings we can support the extensive evidence defending the antioxidant capacity of seminal Zn to yield various benefits in sperm including reduction of MDA and decreasing of DNA fragmentation [10]. Therefore, Zn may be useful in idiopathic oligoasthenoteratozoospermia in reducing oxidative stress and the associated sperm membrane and DNA damage [10].

Se is also an essential trace element that plays an important role in a number of physiological processes including human reproduction [22]. This element becomes crucial for maintaining normal spermatogenesis and male fertility [23]. In our study, we reported a slight increase of seminal Se in controls compared to infertile groups. This was in agreement with the observations of Saaranen et al. [24] and Akinloye et al. [9] who showed a significant decrease of seminal Se levels in asthenozoospermics and oligozoospermics. Takasaki et al. [25] did not find any difference in seminal Se amounts between fertile and infertile men. Meanwhile, a follow-up study 4.5-5 years after the initial assay revealed that low Se levels (< 35 ng/ml) were associated with male infertility.

Our findings sustain the data supporting the negative influence of low seminal Se levels on the number of spermatozoa and sperm motility [9,10]. This evidence can be confirmed by the correlation of the seminal Se and sperm motility observed in our investigation. The association of Se with a protein present in the tail of spermatozoa isolated from bulls and rats [26] and its localization in the mitochondria capsule protein of the midpiece [27] of spermatozoa are possible indications of its importance in sperm motility and male infertility.

Decreased Se concentrations in seminal plasma of infertile men were accompanied by increased levels of seminal MDA of the same patients. In this respect, the diminution of Se activity as an antioxidant can explain the elevated lipid peroxidation in infertile patients. In effect, the only biochemical role of Se in mammals depends on its presence as a seleno-cysteine residue at each of the four catalytic site of the enzyme "GPX" [9]. GPX plays a crucial role in the antioxidant defences of the epididymis and the ejaculated spermatozoa [27].

In addition to the impaired Zn and Se concentrations in seminal plasma of infertile men, we reported also decreased seminal GSHt, GSSG and GSHr levels in the three abnormal groups. Oshendorf et al. [28] found a moderate reduction of GSH in oligozoospermic compared to normozoospermic men, while other studies found GSH levels below the limit of detection (< 2.5 μM) in seminal plasma of oligozoospermics, or GSH levels to be significantly reduced in seminal plasma of infertile males compared to those of fertile ones [1,3]. Even GSH therapy was found to improve the semen quality [29]. Our results provide evidence that higher levels of GSH in seminal plasma seem to play a role in protection against oxidative damage and to improve the sperm motility and morphology. While stating that, other studies could not observe any difference in GSH concentration between fertile and infertile men [8] and this may be due to the contribution of spermatozoal ROS leading to the up-regulation of thiol synthesis in order to protect sperm from oxidative damage. Raijmakers et al. [30] also reported that median levels of seminal GSH were significantly lower in infertile males as compared to normozoospermics, and found a positive association between seminal GSH level and sperm morphology and motility. In our investigation, we found that higher GSHt, GSSG and GSHr levels in seminal plasma were associated with a higher quality of sperm motility and count, however lower GSSG levels were associated with a higher degree of spermatozoa with abnormal morphology and immobility. These findings were compatible with that observed by Bhardwaj A et al. [12] and Eskiocak S et al. [31].

Accordingly; we can suggest that the loss of sperm motility in asthenozoospermic samples may result from the over-oxidation of sperm sulphydryl. GSH displays maximal staining in the mid-piece and tail region, which are important regions for motility of spermatozoa [31]. These results imply that seminal plasma GSH levels may play a role in the protection against oxidative damage by reducing lipid peroxidation on sperm membrane. It could therefore be proposed that the concentration of GSH could be used as a chemical parameter to assess male fertility. The beneficial role of GSH to minimize oxidative damage to the spermatozoa can make it a suitable candidate for therapeutic usage in the treatment of male infertility.

Altered antioxidant status was observed in the seminal plasma, which was collected during our study from infertile men. This might cause oxidative damage and makes the sperm highly susceptible to lipid peroxidation. MDA production reflects the peroxidation of membrane polyunsaturated phospholipids [32,33]. Our results established a significant increase in the amount of seminal MDA in abnormal groups compared to normozoospermics. Accordingly, lipid peroxidative degradation of sperm membrane integrity may be held responsible for abnormal sperm motility, concentration and morphology. Our results were corroborative with Hesham et al. [3] and Ben Abdallah F et al. [34] who reported that MDA content was elevated in oligozoospermic and asthenozoospermic groups. However, there was controversy about seminal MDA activity and sperm quality. We showed that higher seminal MDA levels were negatively associated with sperm motility and sperm count. Nevertheless, a positive relationship was observed between increased lipid peroxidation and the abnormal sperm morphology, which was compatible with the findings of Suleiman et al. [35]. Increased MDA levels in seminal plasma of abnormal groups could represent the pathological effects lipid peroxidation has on the spermatozoa membrane and consequently on sperm motility and viability.

## Conclusions

In summary, associations of seminal Zn, Se and GSH with other parameters of semen quality indicate that the decrease of seminal antioxidants can be a risk factor for sperm abnormality and idiopathic male infertility. It was also shown that increased MDA levels in the abnormal groups could represent the pathological effects of lipid peroxidation on sperm function. These data suggested that routine determination of antioxidant status during infertility investigation is recommended. Furthermore, future research should focus on enzymatic and non-enzymatic antioxidants; genetic susceptibility and their repercussions on semen quality.

## Abbreviations

ROS: Reactive Oxygen Species; PUFAs: Polyunsaturated Fatty Acids; MDA: Malondialdehyde Acid; SOD: Superoxide Dismutase; GPX: Glutathione peroxidase; GRD: Glutathione peroxidase; GSH: Glutathione; Zn: Zinc; DNA: Desoxirubonucleic Acid; Se: Selenium; GSHt: Total glutathione; GSHr: Reduced glutathione; GSSG: Oxidized glutathione; Normo: Normozoospermic men; Astheno: Asthenozoospermic men; Oligo: Oligozooseprmic men; Terato: Teratozoospermic men; AAS: Atomic Absorption Spectroscopy; TBA: Thiobarbituric Acid; SD: Standard Deviation; Max: Maximum; Min: Minimum; NS: Not Significant.

## Competing interests

The authors declare that they have no competing interests.

## Authors' contributions

All the authors made substantial contributions to the design and conception of the study. Particularly, FA wrote the manuscript, contributed to the analysis and interpretation of the data. AK conceived of the study, and participated in its design and coordination and helped to draft the manuscript. MA contributed to the development of the protocol and study instruments. All the authors have been involved in drafting and revising the manuscript, have read, and approved the final manuscript.

## Pre-publication history

The pre-publication history for this paper can be accessed here:

http://www.biomedcentral.com/1471-2490/12/6/prepub
